# Rac1-mediated cytoskeleton rearrangements induced by intersectin-1s deficiency promotes lung cancer cell proliferation, migration and metastasis

**DOI:** 10.1186/s12943-016-0543-1

**Published:** 2016-09-14

**Authors:** Niranjan Jeganathan, Dan Predescu, Jin Zhang, Fei Sha, Cristina Bardita, Monal Patel, Stephen Wood, Jeffrey A. Borgia, Robert A. Balk, Sanda Predescu

**Affiliations:** 1 Division of Pulmonary and Critical Care Medicine, Rush University Medical Center and Rush Medical College, 1750 W. Harrison Street, 299 Jelke South Center, Chicago, IL 60612 USA; 2 Department of Pharmacology and Division of Pulmonary and Critical Care Medicine, Rush University, 1750 W. Harrison Street, 1415 Jelke, Chicago, IL 60612 USA; 3 Department of Pharmacology, Rush University, 1750 W. Harrison Street, 1533 Jelke, Chicago, IL 60612 USA; 4 Department of Pharmacology, Rush University, 1750 W. Harrison Street, 1537 Jelke, Chicago, IL 60612 USA; 5 Department of Immunology, Rush University, 1735 W. Harrison Street, 663 Cohn, Chicago, IL 60612 USA; 6 Department of Biochemistry, Rush University, 1750 W. Harrison Street, 1415 Jelke, Chicago, IL 60612 USA; 7 Division of Pulmonary and Critical Care Medicine, Rush University Medical Center and Rush Medical College, 1750 W. Harrison Street, 293 Jelke, Chicago, IL 60612 USA; 8 Department of Pharmacology and Division of Pulmonary and Critical Care Medicine, Rush University Medical Center and Rush Medical College, 1750 W. Harrison Street, 1535 Jelke, Chicago, IL 60612 USA

**Keywords:** Lung cancer, Cell migration, Cell proliferation, E3 ubiquitin ligase, Rac1, RhoA, Tumor metastasis, Intersectin-1s, mSos1, Eps8, Cbl

## Abstract

**Background:**

The mechanisms involved in lung cancer (LC) progression are poorly understood making discovery of successful therapies difficult. Adaptor proteins play a crucial role in cancer as they link cell surface receptors to specific intracellular pathways. Intersectin-1s (ITSN-1s) is an important multidomain adaptor protein implicated in the pathophysiology of numerous pulmonary diseases. To date, the role of ITSN-1s in LC has not been studied.

**Methods:**

Human LC cells, human LC tissue and A549 LC cells stable transfected with myc-ITSN-1s construct (A549 + ITSN-1s) were used in correlation with biochemical, molecular biology and morphological studies. In addition scratch assay with time lapse microscopy and in vivo xenograft tumor and mouse metastasis assays were performed.

**Results:**

ITSN-1s, a prevalent protein of lung tissue, is significantly downregulated in human LC cells and LC tissue. Restoring ITSN-1s protein level decreases LC cell proliferation and clonogenic potential. In vivo studies indicate that immunodeficient mice injected with A549 + ITSN-1s cells develop less and smaller metastatic tumors compared to mice injected with A549 cells. Our studies also show that restoring ITSN-1s protein level increases the interaction between Cbl E3 ubiquitin ligase and Eps8 resulting in enhanced ubiquitination of the Eps8 oncoprotein. Subsequently, downstream unproductive assembly of the Eps8-mSos1 complex leads to impaired activation of the small GTPase Rac1. Impaired Rac1 activation mediated by ITSN-1s reorganizes the cytoskeleton (increased thick actin bundles and focal adhesion (FA) complexes as well as collapse of the vimentin filament network) in favor of decreased LC cell migration and metastasis.

**Conclusion:**

ITSN-1s induced Eps8 ubiquitination and impaired Eps8-mSos1 complex formation, leading to impaired activation of Rac1, is a novel signaling mechanism crucial for abolishing the progression and metastatic potential of LC cells.

**Electronic supplementary material:**

The online version of this article (doi:10.1186/s12943-016-0543-1) contains supplementary material, which is available to authorized users.

## Background

Metastasis is the primary cause of death in most human cancers, including LC [1]. Metastasis is a multistep process in which cells from the primary tumor migrate through the extracellular matrix (ECM), enter the circulation through tumor angiogenesis and disseminate to distant sites where proliferation begins again [2, 3]. The signal transduction initiated by receptor tyrosine kinases (RTK) plays a pivotal role in the regulation of proliferation and migration of cancer [4]. In most signaling pathways the adaptor proteins link cell surface receptors, including RTK, to specific intracellular pathways regulating the activation status of kinases and controlling the cross-talk between signaling cascades [5]. ITSN-1s is one such protein that plays a crucial role in intracellular signal transduction, linking activated receptors to downstream targets by binding to specific phospho-tyrosine-containing sequences and proline-rich motifs [6–8]. Studies have shown that ITSN-1s is critical for endocytosis of cell surface receptors [9]. Endocytic deficiency induced by ITSN-1s knockdown alters the Smad2/3-Erk1/2 signaling balance downstream of transforming growth factor receptor type I leading to proliferation and neo-vascularization of lung endothelial cells [9]. Due to its multimodular structure, ITSN-1s is involved in multiple protein-protein interactions with a pivotal role in cell growth, apoptosis, cell cycle regulation, DNA damage repair and innate immune response [10, 11].

There are two ITSN genes in humans (ITSN-1 and ITSN-2), each encoding a short (ITSN-s) and long (ITSN-l) isoform due to mRNA alternative splicing of each gene [12]. ITSN-s contains two N-terminal Eps15 homology domains (EH1 and EH2), a coiled-coil (CC) region, and five Src homology 3 domains (SH3A-E). The EH domains promote interaction with Asp-Pro-Phe sequences that are present in numerous endocytic adaptor proteins and the SH3 domains recognize proline-rich domains present in a variety of cytoskeletal and signaling proteins [13]. ITSN-l in addition has an extended C-terminus which comprises the Dbl and pleckstrin (DH-PH) domains with guanine nucleotide exchange factor (GEF) activity for Rho GTPases, especially Cdc42 [14]. ITSN-1l is specific to the brain and is absent in lung tissue whereas ITSN-1s and ITSN-2s and ITSN-2l are expressed ubiquitously [15]. ITSN-1s especially is highly prevalent in lung tissue and plays a significant role in the pathogenesis of pulmonary diseases such as pulmonary hypertension and acute lung injury [9, 16, 17]. ITSN-1s interacts with the Cbl E3 ubiquitin ligase with an essential role in tumorigenesis and metastasis [7, 8, 18]. E3 ubiquitin ligases are often deregulated in human cancers, including LC, and their deregulation has been shown to contribute to cancer development [19]. In addition, ITSN-1s via its SH3 domains interact with mSos1 which is a GEF for the GTPase proteins Ras and Rac1 and is also an Eps8-interacting protein [20]. Eps8 interacts with the actin cytoskeleton to facilitate Rac1 localization and subsequent cell migration [21]. In addition, ITSN-1s binds to CdGAP (GTPase-activating protein) via its SH3 domains with activity towards Cdc42 and Rac1 [22, 23].

Clinical evidence suggests that ITSN-1s may also be important in cancer. The ITSN-1 gene is present on chromosome 21 in the Down syndrome critical region [12]. As a result, ITSN-1 mRNA and protein levels are elevated in these patients [24]. In a cohort prospective registry study these patients had a significantly lower incidence of LC compared to the age matched general population [25].

Based on this background, we hypothesized that ITSN-1s may play a role in LC progression. Our novel findings demonstrate that ITSN-1s is significantly downregulated in human LC cells and LC tissue. Restoring ITSN-1s protein level increases ubiquitination of Eps8 oncoprotein and impairs Eps8-mSos1 interaction, leading to decreased activation of Rac1 GTPase resulting in cytoskeleton rearrangements capable of abolishing the progression and metastatic potential of LC cells.

## Methods

### Cell culture and transfection

A549 cells were grown in HAM’s F12 medium, containing 10 % FBS, 1 % 1-Alanyl-L-glutamine, and 1 % antimycotic, at 37 °C in a 5 % CO_2_ incubator. Bronchial cells were grown in airway epithelial basal cell medium (0.25 % HLL, 3 % L-glutamine, 0.4 % Extract P, 1 % airway epithelial cell supplement). Subconfluent A549 cells were transfected with a myc-tagged full-length ITSN-1s DNA cloned into the mammalian vector pcDNA3.1Myc/His (GeneCopoeia™, Rockville, MD), using 7:2 ratio of Fugene HD transfection reagent (μl) to DNA (μg) [26]. For stable transfection, ITSN-1s transiently transfected cells were grown in 100 cm^2^ dishes with 1 mg/ml G418. HAM-F’12 medium containing 1 mg/ml G418 was replenished every other day for about 2 weeks until stable transfected A549 cells began to grow. At that point the concentration of G418 in HAM’s F12 medium was switched to 800 μg/ml for a week and then maintained at 500 μg/ml. For siRNA transfection, a set of 4 siRNA targeting Cbl gene, control siRNA and Transfection Reagent from Dharmacon (Lafayette, Co) were used; 0.1×10^6^ cells in 2 ml complete medium without antibiotics were seeded in 6-well plates and grown to 70 % confluence prior to transfection. Control siRNA and Cbl siRNA (5 μM) were diluted in transfection medium (4 μl) as per manufacturer’s instructions and added dropwise over the cells. Transfected cells were incubated at 37 °C in a 5 % CO_2_ for 8 h without changing medium. Then the medium was replaced with normal growth medium. Optimal siRNA/transfection reagent ratio and transfection time were determined in pilot studies.

Specific Antibodies (Ab) were as follows: ITSN-1, Eps8 (BD Biosciences, San Jose, CA); mSos1, Rac1, Cbl, control IgG, Ub, vinculin, vimentin (Santa Cruz Biotechnology, Santa Cruz, CA); ITSN-1, Actin (Sigma-Aldrich, St. Louis, MO). All fluorophor-conjugated Abs and the Prolong Antifade reagent were from Molecular Probes (Eugene, OR).

### Human LC specimens

LC tissue micro array (TMA) with 20 cases of lung adenocarcinoma and 20 cases of normal lung tissue was obtained from US Biomax (Rockville, MD). LC cell lysates, frozen lung tissue specimens and normal lung controls were provided by Dr. Jeffrey Borgia, Rush University Medical Center (Chicago, IL). Primary lung adenocarcinoma samples (*n* = 10) in paraffin-embedded blocks were obtained from the Department of Pathology, Rush University Medical Center (Chicago, IL). The studies were approved by the Rush Human Subject Committee. Informed consent from the patients was obtained in all cases. Lung histological sections were analyzed in each case to confirm the diagnosis. The LC cell lines were derived from lung tissue as follows: A549, lung carcinoma; H358, bronchioalveolar carcinoma; H1703, squamous cell carcinoma; H1437, adenocarcinoma (stage 1; pleural effusion); H2009, adenocarcinoma (stage 4; lymph node).

### Quantitative PCR

Lung tissues and cells were lysed using the Qiagen QIA Shredder homogenizer; total RNA was isolated using the RNeasy Mini RNA isolation kit (Qiagen, Valencia, CA). The cDNA was synthesized from 2 μg RNA using High-Capacity cDNA Reverse Transcription Kit (Applied Biosystems, Carlsbad, CA). RT-PCR was performed on an Applied Biosystems 7900HT machine [17]. The relative mRNA levels were normalized to housekeeping gene cyclophillin, and determined by calculating the ΔΔCt value as per manufacturer’s guidelines.

### Soft agar assay

Anchorage-independent growth was determined by assaying colony formation in soft agar [28]. Briefly, 1 × 10^5^ A549 and A549 + ITSN-1s cells resuspended in 5 ml HAM’s F12 medium (containing 10 % FBS and 0.28 % Difco Nobal Agar) were plated in triplicates onto 35 mm dishes over a 2.5 ml layer of solidified HAM’s F12/10 % FBS and 0.58 % agarose. The cells were incubated in humidified 5 % CO_2_, 95 % air, at 37 °C and fed by adding 2 ml of HAM’s F12/10 % FBS every 2–3 days. Thirty days after seeding, colonies were stained with 0.01 % crystal violet dye and counted. Pictures were taken by Alpha Imager HP digital scanner.

### MTT assay

A549 cells and A549 + ITSN-1s cells were subjected to MTT cell proliferation [17]. Briefly, triplicate aliquots of cells (10^6^ cells suspended in 100 μl complete medium) were serially diluted in medium, onto a 96-well plate. After 48 h, 10 μl MTT Reagent were added to each well. After 5 h incubation, 100 μl of detergent was added, the plate was covered and kept in the dark at room temperature (RT) overnight. The next day the absorbance at 570 nm was determined using a Dynex microplate reader. Parallel triplicate experiments using non-treated cells were performed; cells were counted using a hemocytometer and a growth curve was generated to relate the OD^570^ values to the cell number per well.

### Immunoprecipitation and western blot

Cells were lysed in a buffer containing 50 mM Tris-HCl-pH8.0, 150 mM NaCl, 1 % NP-40, 1 mM Na_3_VO_4_, 1 mM PMSF, and protease and phosphatase inhibitors for 2 h at 4 °C. Lysates were centrifuged (45 min, at 45000 rpm, at 4 °C) and stored at −20 °C. Protein content was determined using Micro Bicinchoninic acid assay. For immunoprecipitation (IP), cell lysates (500 μg of total protein) were pre-cleared using 25 μl protein A/G slurry for 30 min at RT. The supernatants were then incubated with 3 μg mSos1, Cbl or isotype matched IgG Abs for 1 h at RT. 40 μl of protein A/G slurry (Santa Cruz Biotechnology, Santa Cruz, CA) were then added to the supernatants and incubated at 4 °C overnight. The bound proteins were resolved on 5–20 % SDS-PAGE. Western blot (WB) was performed using primary Abs and appropriate secondary Abs [26]. TrueBlot® ULTRA secondary Ab (Rockland Immunochemicals, Limerick, PA) was used for Rac1 to avoid background from the light chain band. For detection of Eps8 ubiquitination by WB, 5 mM N-ethylmaleimide was added in the IP buffer to prevent the cleavage of polyubiquitin chains [9].

### Analysis of soluble to insoluble vimentin ratio

A549 cells and A549 + ITSN-1s cells were grown under similar conditions, trypsinized and counted. Equal number of cells were lysed and centrifuged for 5 min at 10,000xg. The pellet (vimentin full length filaments) and 60 μg total protein of supernatant (collapsed vimentin filaments) were solubilized in equal volume of Laemmli sample buffer, denatured by boiling, and equal volumes were resolved on 5–20 % SDS-PAGE and analyzed by WB with vimentin Ab [29].

### Immunofluorescence and immunohistochemistry

Immunofluorescent and phalloidin staining of cells grown on coverslips was performed [30]. Briefly, cells were seeded on non-collagen coated coverslips in 6-well plates. Cells were washed three times with cold PBS, followed by fixation and permeabilization with methanol at −20 °C for 5 min. Methanol was immediately removed and blocking buffer (PBS with 1 % BSA) was added for 45 min at RT. Following removal of blocking buffer, cells were incubated with primary Ab (ITSN-1 Ab (1:250), vinculin Ab (1:250) or vimentin Ab (1:250) in 0.1 % BSA/PBS) for 1 h at RT. The cells were washed with 0.1 % BSA in PBS three times for 10 min each. Coverslips were then incubated with appropriate secondary Ab (at dilution 1:500 in 0.1 % BSA/PBS) for 1 h, protected from light, at RT followed by washing as above. For phalloidin Alexa Flour (AF) 488 staining, cells were fixed in 3.7 % paraformaldehyde in PBS for 15 min at RT, permeabilized in 0.1 % Triton X-100 for 5 min on ice, and stained with phalloidin AF488 for 30 min at RT. Cells were mounted with Prolong Antifade reagent. For morphometry, the number of vinculin clusters per 250 μm^2^ was counted in 3 randomly chosen areas along the cell perimeter and was averaged from different experiments performed in triplicates. At least 50 cells were counted per experiment. Micrographs were taken with a Zeiss AxioImager M1 microscope equipped with a digital camera. All images used for quantification were acquired using identical parameters per experiment.

Immunohistochemistry (IHC) on paraffin-embedded LC specimens and TMA using the ITSN-1 Ab was performed as previously described by us [17]. ITSN-1s Ab from Sigma-Aldrich (St. Louis, MO) was used for immunofluorescence and IHC.

### Actin polymerization assay

Briefly, cells were washed in PBS and collected into 1.5 ml Eppendorf tubes. Cell pellets were then resuspended in 100 μl of lysis buffer I (20 mM Hepes-NaOH, pH 7.2, 100 mM NaCl, 1 mM sodium orthovanadate, 50 mM NaF, 1 % Triton X-100, protease inhibitors) for 1 h and centrifuged for 20 min at 22,000 rpm [30]. Supernatants were saved as G-actin fractions; the pellet was resuspended in lysis buffer II (15 mM Hepes-NaOH, pH 7.5, 0.15 mM NaCl, 1 % Triton X-100, 1 % sodium deoxycholate, 0.1 % SDS, 10 mM EDTA, 1 mM dithiothreitol, 1 mM sodium orthovanadate, protease inhibitors) for 1 h and then centrifuged at 45,000 rpm for 40 min. The ensuing supernatants represented the F-actin fractions. Equal protein amounts were loaded onto 4–12 % SDS-PAGE. Actin was detected by immunoblotting with actin Ab and quantified by densitometry.

### Scratch assay

Cells were seeded into 12-well plates and grown to 90–100 % confluence. A sterilized micro-pipette tip was used to generate a scratch across the cell layer. Cells at multiple points along the scratch were imaged every 15 min over a 48 h period using time-lapse microscopy using a Zeiss AxioVert Z1 microscope. Images were captured at 100× magnification. Cell migration measurements were made using NIH imageJ software.

### Mouse metastasis and xenograft tumor assays

Protocols were approved by the Institutional Animal Care and Use Committee and Institutional Biosafety Committee of Rush University. Five week old immunodeficient male mice (Jackson Labs, ME) were used. For metastasis assay, mice were injected retro-orbitally with 2 × 10^**6**^ cells in 0.1 ml PBS. After 12 weeks mice were sacrificed. The lungs were removed, fixed in 4 % formaldehyde/PBS, paraffin-embedded, dissected and stained with hematoxylin and eosin (H&E) (27). For the xenograft tumor assay, 10^7^ cells resuspended in PBS were mixed with matrigel (BD Bioscience, Bedford, MA) at a 1:1 volume ratio in 200 μl of medium without serum and injected subcutaneously into the flanks of mice [31]. After 4 weeks, mice were sacrificed and the tumor was resected. Pictures were taken and the tumor area was measured NIH imageJ software.

### Cdc42/Rac1/RhoA activation assay

Cell lysates prepared in ice-cold lysis buffer were centrifuged (10,000 g, 4 °C for 1 min) to remove cell debris and then incubated with 40 μl of p21 activated kinase (PAK)-GST beads [Cdc42/Rac1 activation kit (Cytoskeleton, Denver, CO)] or 50 ml of Rhotekin-GST beads for RhoA for 60 min at 4 °C, as per manufacturer’s instructions. Beads were washed and boiled for 2 min in 20 μl of Laemmli sample buffer; samples were run in parallel with total cell lysates and GTP/GDP control, and WB performed with Cdc42/Rac1/RhoA Ab. Activation of Cdc42/Rac1/RhoA in control and transfected cells was determined by densitometry.

### Statistical analysis

All findings were confirmed in three to five different experiments performed under identical conditions. Images were acquired using identical parameters. Densitometry analysis was performed using NIH ImageJ software. Data are expressed as mean or median ± SE. Comparisons were made using unpaired student’s *t*-test. *P* values less than 0.05 were considered statistically significant.

## Results

### ITSN-1s protein and mRNA levels are downregulated in LC cells and tissues

**Fig. 1:**
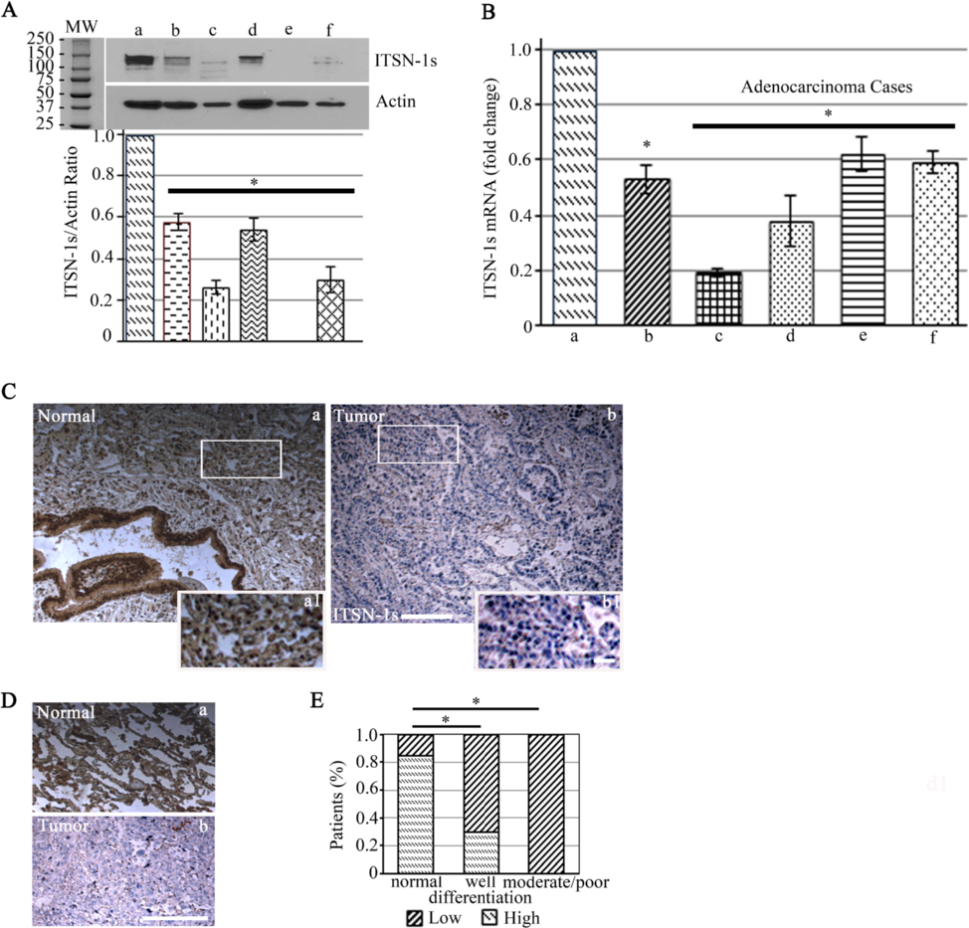
ITSN-1s protein and mRNA levels are decreased in LC patients. **a** WB using ITSN-1 Ab of cell and lung tissue lysates resolved by SDS PAGE (70 μg total protein/lane). Human LC cells (*b - f*) were compared to control bronchial cells (a). The cell lines were as follows: b- A549, c- H358, d- H1703, e- H1437, f- H2009; MW – molecular weight. Data are shown as ITSN-1s/actin ratio. The bands between MW 100–150 were included in densitometry analysis. **b** qPCR analyses of ITSN-1s mRNA levels in A549 cells (*b*) and lung adenocarcinoma cases (c–f) compared to controls (bronchial cells and normal tissue) (a). Data are shown as percentage decrease by comparison to controls. Error bar represents mean ± SE; *p < 0.05. The above data are representative of 3 independent experiments performed under identical experimental conditions. **c** Representative ITSN-1s IHC staining of paraffin-embedded adjacent normal lung tissues (*a, a1*) and lung adenocarcinoma tissue (*b, b1*). **d** Representative ITSN-1s IHC staining of TMA specimens from normal lung (*a*) and lung adenocarcinoma (*b*). **e** The percentage of patients with high and low ITSN-1s protein level were calculated in 3 groups: normal, well-differentiated and moderately or poorly differentiated. Images were acquired using identical parameters. Bars: 20 μm (*a, b*); 5 μm (*a1, b1*)

To address whether ITSN plays a role in LC, we examined ITSN-1s protein level in human LC cells by WB with ITSN-1 Ab compared to human bronchial cells (Fig. 1a). Downregulation of ITSN-1s protein level was consistent for all LC cell lines (Fig. 1a, lanes b – f vs. a). Densitometry indicated that the extent of downregulation ranged from 42 % to undetectable levels in H1437 adenocarcinoma cells (Fig. 1a, e). To determine if downregulation of ITSN-1s is due to inhibition of transcription or post-translational modifications, qPCR analyses were performed. ITSN-1s mRNA levels were assessed in A549 cells compared to bronchial cells, and in adenocarcinoma tissue (Table 1), compared to non-LC tissue (Fig. 1b). Similar to protein level, ITSN-1s mRNA level was decreased in LC by 38 to 81 %.

**Table 1:** Characteristics of the LC patients

Case	Location	Differentiation	T	N	M	Stage	Sex	Age
c	LLL	Poorly	2	2	0	3a	F	64
d	RLL	Moderately	2	2	0	3a	M	69
e	RUL	Well	1	0	0	1a	F	67
f	LLL	Well	2	0	0	1b	M	67

All cases were lung adenocarcinoma. The cases consisted of poorly to well-differentiated samples from patients with early to advanced LC (stage 1 to 3). LLL – left lower lobe, RUL – right upper lobe, RLL – right lower lobe; T – tumor size, N – lymph node, M – metastasis, F - female, M – male, stage of LC is based on TNM staging system [27]

To further confirm this finding we examined the level of ITSN-1s in 10 lung adenocarcinoma tissue samples along with adjacent normal tissue. IHC analyses revealed high ITSN-1s immunostaining in the normal bronchial epithelial cells and adjacent normal alveoli (Fig. 1c, a, a1) but much weaker or even undetectable levels in some tumors (Fig. 1c, b, b1). The level of ITSN-1s was reduced by more than 50 % compared to normal areas in 8 of the adenocarcinoma samples examined. In addition, we evaluated a TMA slide comprising 20 lung adenocarcinoma samples: 10 grade 1 (well differentiated) and 10 grades 2 or 3 (moderate to poorly differentiated) and 20 normal tissue samples. Each TMA spot was examined and graded as having high (Fig. 1d, a) or low ITSN-1s (Fig. 1d, b) protein level. The presence of at least 25 % of cancer cells staining positive (intensity of a minimum of 2 on a scale of 1 to 3 [32, 33]) within a patient sample was considered high ITSN-1s protein level. A high ITSN-1s protein level was noted in 85 % of normal tissue, 30 % of grade 1 LC tissue and none of the grades 2 or 3 LC tissue (Fig. 1e). Altogether these findings provide strong evidence that ITSN-1s protein level is downregulated in LC.

**Fig. 2:**
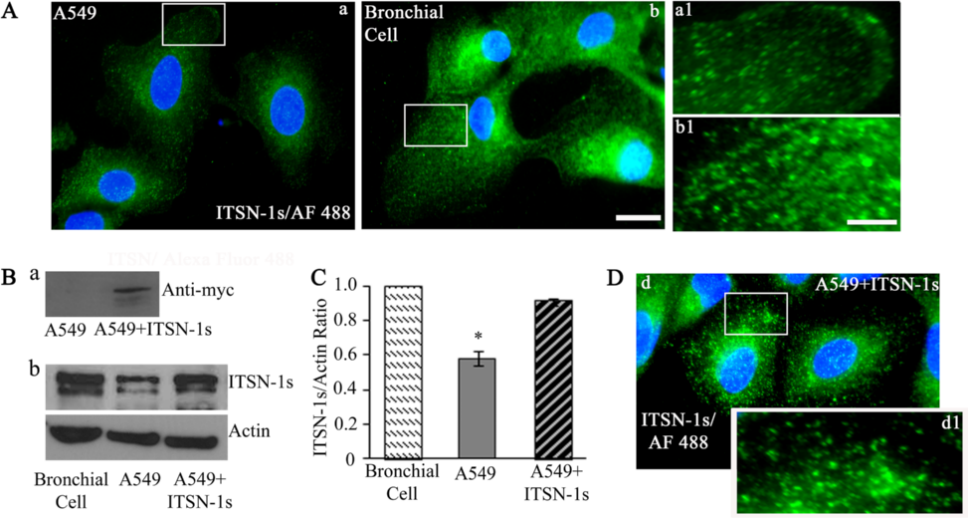
Subcellular distribution of ITSN-1s in LC cells. **a** Immunofluorescent staining with ITSN-1 Ab followed by AF488 Ab in A549 cells (*a, a1*) compared to bronchial cells (*b, b1*). **b** ITSN-1s protein expression was restored by stable transfection in A549 cells: myc-ITSN-1s protein level is detected via myc Ab in transfected A549 cells (*a*); lysates of bronchial cells, A549 cells and myc-ITSN-1s transfected A549 cells (A549 + ITSN-1s) were assessed for ITSN-1s protein level by WB using ITSN-1s Ab; actin used as loading control (*b*). **c** ITSN-1s protein level was quantified by densitometry. **d** Immunofluorescent staining of A549 + ITSN-1s cells with ITSN-1 Ab shows a similar subcellular distribution but increased intensity in A549 + ITSN-1s cells compared to A549 cells (*d, d1*). Error bar represents mean ± SE; *p < 0.05. Data are representative of 3 independent experiments performed under identical experimental conditions. Images were acquired using identical parameters. Bars: 20 μm (*a, b, d*); 5 μm (*a1, b1, d1*)

Next, we examined the subcellular distribution of ITSN-1s in A549 cells compared to bronchial cells. Immunofluorescent staining using ITSN-1 AF 488-conjugated reporter Abs showed a wide subcellular distribution of ITSN-1s in bronchial cells; the strong punctate pattern at the plasma membrane and in the cytoplasm as well as in the perinuclear area is consistent with ITSN-1s’ association with the endocytic and Golgi vesicles (Fig. 2a, b, b1), as previously reported in other cell types [26, 34]. A549 cells revealed a similar subcellular distribution of ITSN-1s (Fig. 2a, a, a1), but a decreased staining intensity, consistent with downregulation of ITSN-1s protein level in LC.

To determine the impact of ITSN-1s downregulation on LC, we first restored ITSN-1s protein level in A549 cells using myc-ITSN-1s. To improve and to preserve the level of ITSN-1s long term, we created stable transfected myc-ITSN-1s A549 cells (A549 + ITSN-1s; Fig. 2b). Western blot using myc Ab (Fig. 2b, a) and ITSN-1s Ab (Fig. 2b, b) demonstrated successful transfection. Densitometry indicated restoration of ITSN-1s protein level to 91 % of normal bronchial cells (Fig. 2c). Immunoflorescence staining (ITSN-1s/AF 488 Ab) of A549 + ITSN-1s cells (Fig. 2d, d, d1) compared to A549 cells (Fig. 2a, a, a1), demonstrated a similar subcellular distribution but increased staining intensity, consistent with restoration of ITSN-1s protein level.

### ITSN-1s impairs LC cell proliferation, anchorage-independent growth and tumor growth

**Fig. 3:**
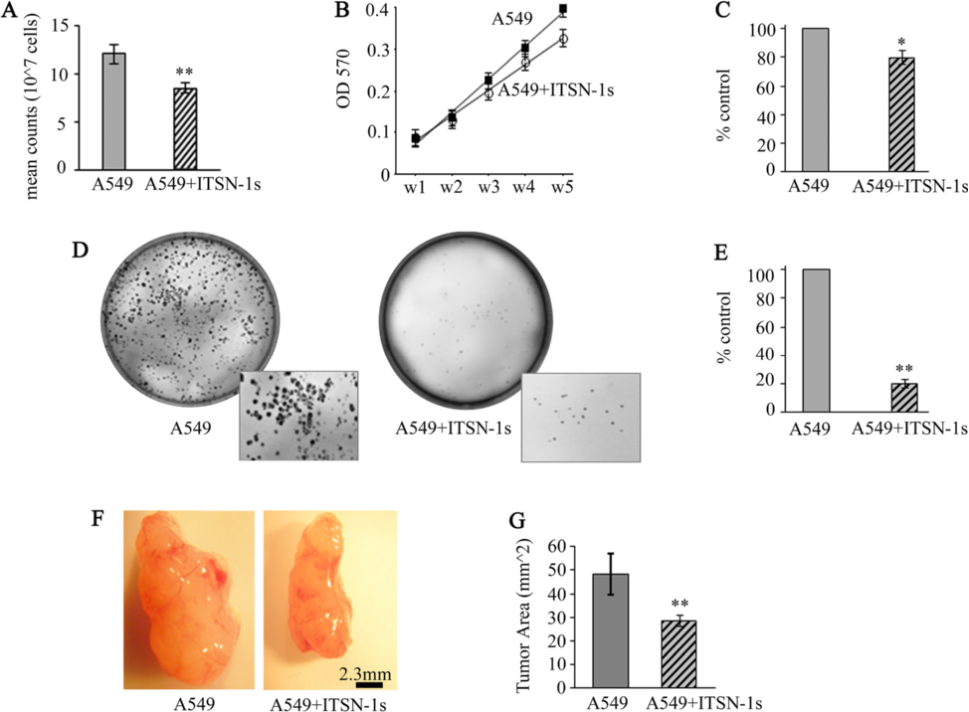
ITSN-1s impairs LC cell proliferation and growth. **a** A549 and A549 + ITSN cells (10^7^) were seeded in 100 cm^2^ plates, cultured for five days, trypsinized and counted. **b** Serial dilutions of A549 and A549 + ITSN-1s cells seeded in 96-well plates were subjected to the MTT assay. Cell proliferation was assessed by absorbance at 570 nm; (w-well). **c** Three points on the linear part of the proliferation curve were used to evaluate the extent of cell growth inhibition. Data are shown as percentage of control. **d** A549 and A549 + ITSN-1s cells (5×10^5^) were seeded in 35 mm dishes in media with 0.28 % agar on a base of 0.58 % agar. Colonies began to form 30 days later, and were stained with crystal violet dye. Representative plates are shown. **e** The number of colonies was quantified. Data are shown as percentage of control. Error bar represents mean ± SE; *p < 0.05; **p < 0.01. The above data are representative of 3 independent experiments performed under identical experimental conditions. **f** A549 and A549 + ITSN cells (10^7^) injected into nude mice. Shown are representative tumors that formed 30 days post-injection. **g** Mean tumor area was measured using NIH ImageJ. Error bar represents mean ± SE; (*n* = 3-5 mice); *p < 0.05

Prior studies have shown that ITSN-1s regulates cell proliferation via interaction with Ras [35]. Therefore, we decided to investigate the effects of restoring ITSN-1s protein level on LC cell proliferation. Briefly, A549 and A549 + ITSN-1s cells were seeded in 100 cm^2^ plates in triplicate, cultured for five days and counted. A549 + ITSN-1s cells showed 30 % inhibition of cell proliferation compared to A549 cells (Fig. 3a). A549 and A549 + ITSN-1s cells proliferation was also evaluated by the MTT assay. A growth curve was generated to relate the OD^570^ values to the cell number per well. The OD^570^ values for A549 + ITSN-1s cells were significantly lower compared to A549 cells in all wells of the cell culture plate (Fig. 3b). Three points on the linear part of the proliferation curve were used for quantification of the extent of cell growth inhibition. The results indicated a 20 % inhibition in proliferation of A549 + ITSN-1s cells compared to A549 cells (Fig. 3c).

Since restoring ITSN-1s level impairs A549 cell proliferation, we examined whether ITSN-1s regulates anchorage-independent growth, one of the hallmarks of cell transformation [28]. We performed the soft-agar assay, a validated in vitro assay for detecting malignant transformation [28]. Cells started to form noticeable colonies after 30 days of culture. Although both A549 and A549 + ITSN-1s cells formed colonies, the ability of A549 + ITSN-1s cells to grow independent of anchorage was impaired compared to A549 cells (Fig. 3d); the number of colonies was 80 % lower (Fig. 3e), suggesting that ITSN-1s deficiency is an important component in the anchorage-independent growth of human LC cells.

To gain insight into ITSN-1s’ role in anchorage-independent growth of LC cells in vivo*,* we performed a xenograft tumor assay [31]. Immunodeficient mice were injected subcutaneously with A549 and A549 + ITSN-1s cells. Tumor development and growth were monitored for 4 weeks at which point tumors were resected, photographed (Fig. 3f), and measured. The tumors of mice injected with A549 + ITSN-1s cells were 42 % smaller than the tumors of mice injected with A549 cells (Fig. 3g). Together these studies demonstrate that ITSN-1s restoration in A549 cells significantly imapirs tumor proliferation and anchorage-independent growth.

### ITSN-1s impairs LC cell migration and metastasis

**Fig. 4:**
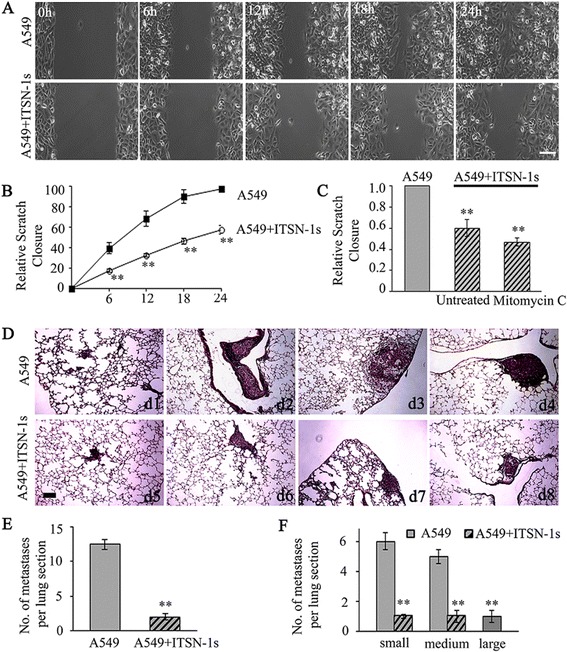
ITSN-1s impairs LC cell migration and metastasis. **a** A549 and A549 + ITSN-1s cell layer was wounded using a sterile micropipette tip and cells at multiple points along the wound edge were imaged every 15 min over a 48 h period using time-lapse microscopy. Bar: 10 μm. **b** Measurements were made using NIH ImageJ. Scratch closure was expressed as a percentage of initial scratch area in each well. **c** Scratch closure in mitomycin C treated and untreated A549 and A549 + ITSN-1s cells were compared. Data are shown as percentage of control. Error bar represents the mean ± SE; **p < 0.01. Data are representative of 3 independent experiments performed under identical experimental conditions. **d** Histological analyses of lung metastasis in immunodeficient mice injected retroorbitally with A549 and A549 + ITSN-1s cells. Bar: 100 μm. **e**, **f** Total number as well as the size of the lung metastasis per section was determined. Data are shown as median ± SE; **p < 0.01 (*n* = 5-7 mice). 5 lung sections per mice were examined using identical parameters

To address whether ITSN-1s deficiency interferes with migration of LC cells, we performed a scratch assay which preserves cell-cell interactions and is able to mimic migration of cells in vivo [36], in conjunction with time-lapse microscopy (Fig. 4a). A549 + ITSN-1s cells showed statistically significant inhibition in scratch closure as early as 3 h. The scratch was completely closed by A549 cells at 24 h, whereas, A549 + ITSN-1s cells closed only 60 % of the scratch (Fig. 4b) at this same time point. The scratch closure is due to both cell proliferation and cell migration into the scratch from the periphery. The impact of either proliferation or migration in scratch closure cannot be determined just based on the images, especially given that the cells are grown to confluence prior to creating the scratch and given that cancer cells migrate collectively in sheets/lumps. To determine the impact of increased ITSN-1s protein level on cell migration independent of cell proliferation, cells grown to confluence were pretreated with 7.5 μg/ml of mitomycin C (Sigma-Aldrich, St. Louis, MO) for 1 h which impaired further cell proliferation efficiently without killing the cells (S1, A). Mitomycin C is a widely used antibiotic because of its mild toxicity and potent antitumor activity. Mitomycin C reacts covalently with DNA, forming crosslinks between the complementary strands of DNA. This prevents separation of the complementary strands and thus inhibits DNA replication [37]. Based on the efficiency of inhibition noted with mitomycin C, we believe the additional cells noted in the scratch at 24 h in mitomycin C treated cells (Additional file 1: Figure S1B, 2 panels on the right) is due to predominantly cell migration of remaining cells from the periphery. Mitomycin C treated A549 + ITSN-1s cells compared to A549 controls significantly delayed the scratch closure (S1, B) and overall impaired cell migration by 52 % (Fig. 4c). This finding demonstrates that ITSN-1s plays a significant inhibitory role in cell migration in addition to its antiproliferative effects.

To further validate this finding we used a mouse metastasis assay. A549 and A549 + ITSN-1s cells were injected into the retro-orbital sinus of immunodeficient mice [16]. After 12 weeks, mice were sacrificed and the lungs were prepared for routine morphological analyses [16]. For evaluation of pulmonary metastasis, lungs were cut in 5 μm sections and stained with H&E. Five sections throughout the whole lung per mice were screened histologically, and the number of metastases was counted and classified based on their size as small (diameter <200 μm), medium (diameter 200–500 μm) or large (diameter >500 μm). A549 and A549 + ITSN-1s injected mice developed tumors in the lung parenchyma (Fig. 4d, d1, d5), pulmonary artery (Fig. 4d, d2, d6), sub-pleura (Fig. 4d, d3, d7) and peribronchial lung (Fig. 4d, d4, d8). Mice injected with A549 cells had significantly more tumors per lung section (median 12.5 ± 0.7 vs. 2 ± 0.47, Fig. 4e), as well as more tumors per each size group (median, small 6 ± 0.56 vs. 1 ± 0.12, medium 5 ± 0.47 vs. 1 ± 0.38, large 1 ± 0.39 vs. 0, Fig. 4f) compared to mice injected with A549 + ITSN-1s cells. Together these findings are consistent with an important role of ITSN-1s in restricting LC proliferation, migration and metastasis.

To determine if ITSN-1s was still present in the emergent tumors, we performed immunofluorescence staining of lung sections with ITSN-1s antibody. Tumors of all size in mice injected with A549 cells showed very low to undetectable levels of staining for ITSN-1s (Additional file 2: Figure S2, A, C). However, mice injected with A549 + ITSN-1s cells showed variability in ITSN-1s expression based on the size of the tumors. Smaller tumors showed higher ITSN-1s staining throughout the tumor (Additional file 2: Figure S2, B, b1). In contrast, larger tumors showed more heterogeneous expression with patches of cells showing high ITSN-1s staining and other patches of cells within the same tumor showing low ITSN-1s staining (Additional file 2: Figure S2, D, d1). This supports the role of ITSN-1s as a tumor suppressor.

### ITSN-1s regulates cellular cytoskeleton organization

**Fig. 5:**
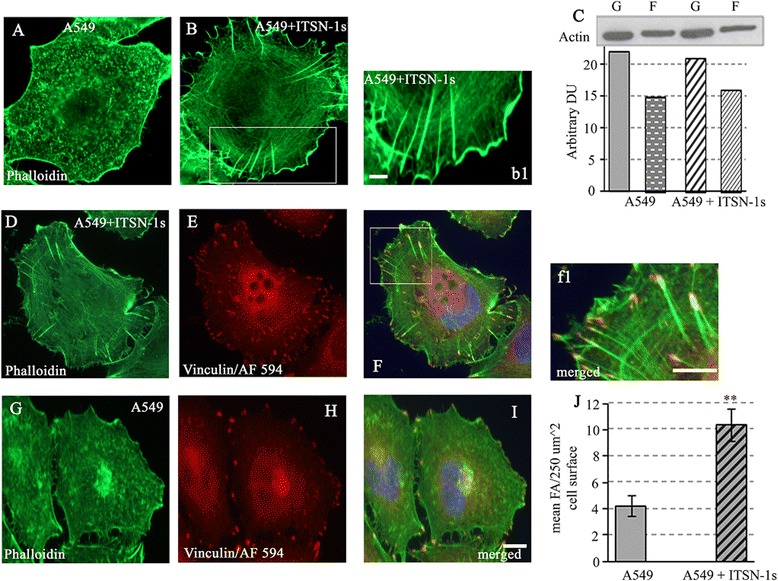
ITSN-1s increases actin bundles and number of FAs. Representative phalloidin staining of A549 (**a**) and A549 + ITSN-1s cells (**b**). Thick actin filaments were often detected in A549 + ITSN-1s wells (*B*, boxed area, *b1*). Cell lysates were subjected to differential centrifugation to obtain the G-actin and F-actin fractions, which were analyzed by WB with actin Ab followed by densitometry (**c**). Representative double fluorescent staining with phalloidin (**d**, **g**) and vinculin/AF594 Ab (**e, h**) of A549 + ITSN-1s and A549 cells respectively. Co-localization of actin/vinculin is significant in A549 + ITSN-1s (**f**, *f1*). Limited co-localization is detected in A549 (**i**). The number of vinculin clusters/250 μm^2^ cell surface was counted (**j**). Data are representative of 3 independent experiments performed under identical experimental conditions. 50 cells per experiment were analyzed. Images were acquired using identical parameters. Bars: 10 μm (A-I); 5 μm (*b1, f1*). Data are shown as mean ± SE; **p < 0.01

Since cancer cell migration and metastasis requires reorganization of the cytoskeleton leading to epithelial-mesenchymal transformation [38], we next addressed whether decreased ITSN-1s protein level may be involved in reorganization of the components of the cytoskeleton to favor migration and metastasis. Phalloidin staining revealed small filaments of actin in A549 cells (Fig. 5a), whereas in A549 + ITSN-1s cells it showed thick bundles of actin pointing towards peripheral attachment points (Fig. 5b, b1). In addition, A549 + ITSN-1s compared to A549 cells showed more spreading and loss of elongated and polarized morphology. Cell fractionation and differential centrifugation of A549 and A549 + ITSN-1s cell lysates followed by WB analyses and densitometry showed no change in the G- and F-actin fractions between the two cell lines (Fig. 5c), suggesting that restoring ITSN-1s protein level does not result in formation of new actin or actin polymerization, but simply reorganizes existing actin to form bundles. Since association/dissociation of the actin cytoskeleton structure with FA proteins determines cell motility and metastasis [39], we performed double actin/vinculin immunofluorescent staining (Fig. 5d – i). A549 + ITSN-1s cells had increased actin-vinculin colocalization (Fig. 5f, f1) and a higher number of vinculin clusters per surface area compared to A549 cells (Fig. 5j). The mean number of vinculin clusters per 250 μm^2^ cell surface was 4.24 ± 0.8 vs. 10.4 ± 1.23 in A549 cells and A549 + ITSN-1s cells respectively.

**Fig. 6:**
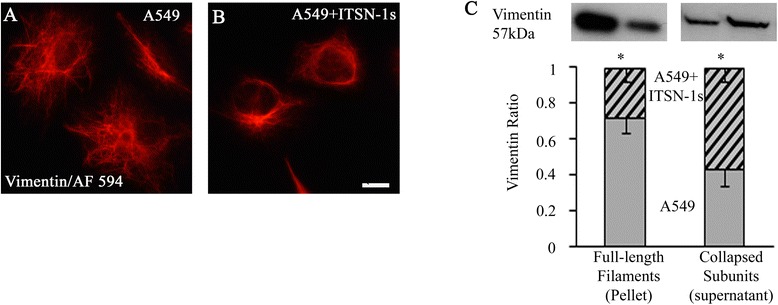
ITSN-1s collapses vimentin filaments. Immunofluorescence staining using vimentin/AF594 Ab demonstrate a widespread vimentin filament network in A549 cells (**a**) and an altered subcellular distribution of vimentin with collapse of vimentin intermediate filaments into clusters of short filaments near the nucleus in A549 + ITSN-1s cells (**b**). Biochemical analysis and densitometry of full length and collapsed vimentin filaments in A549 and A549 + ITSN-1s cells lysates (**c**). Data are representative of 3 independent experiments performed under identical experimental conditions. 50 cells per experiment were analyzed. Images were acquired using identical parameters. Bars: 10 μm (**a, b**). Data are shown as mean ± SE, *p < 0.05

The other component of the cell cytoskeleton involved in metastasis is vimentin intermediate filaments [40]. Immunofluorescence staining and image analyses showed a wide spread vimentin filament network in A549 cells (Fig. 6a). A549 + ITSN-1s cells showed an altered subcellular distribution of vimentin with collapse of vimentin intermediate filaments into clusters of short filaments near the nucleus (Fig. 6b). Next, WB using vimentin Ab was applied on A549 and A549 + ITSN-1s cells lysates as described under Methods. Full-length vimentin filaments which are not soluble in lysis buffer are present in the pellet, whereas the collapsed intermediate and small filaments are soluble and present in the supernatant. Densitometry analyses indicated decreased insoluble vimentin and increased soluble vimentin in A549 + ITSN-1s cells compared to A549 cells (Fig. 6c), consistent with our morphological findings. Based on these findings and previous reports indicating that the collapse of vimentin filaments inhibits cancer progression [41], we concluded that restoring ITSN-1s protein level stabilizes the cells and contributes to decreased motility and metastasis.

### ITSN-1s decreases mSos1-Eps8 interaction leading to impaired activation of Rac1

**Fig. 7:**
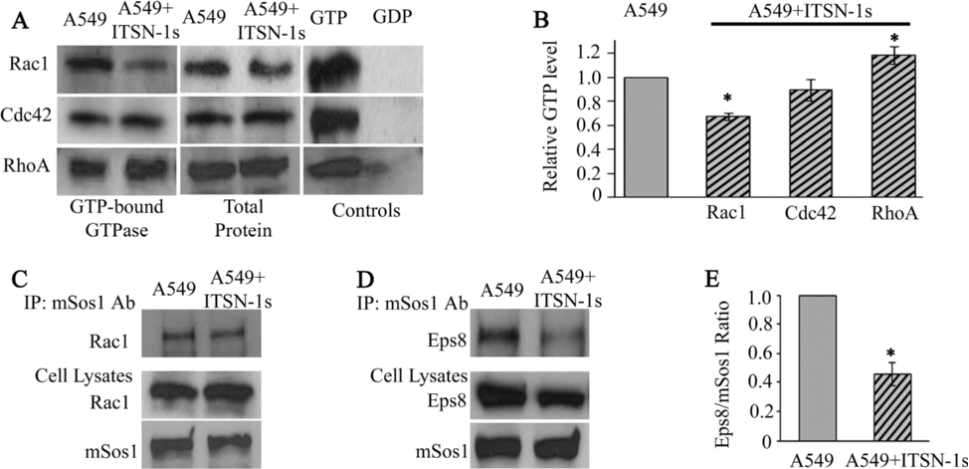
ITSN-1s impairs mSos1-Eps8 leading to deactivation of Rac1. **a** Representative PAK/Rhotekin-GST pull-down assays applied on A549 and A549 + ITSN-1s cell lysates followed by WB with Rac1, Cdc42 and RhoA Abs. **b** Data are shown as percentage of control. Total Rac1, Cdc42, RhoA and GTP/GDP loaded lysates were uses as control. **c**, **d** A549 and A549 + ITSN-1s cell lysates were subjected to IP with mSos1 Ab, followed by WB with Rac1 (**c**, *upper panel*) and Eps8 (**d**, *upper panel*) Abs. The *lower panels* in **c** and **d** illustrate the Rac1, mSos1 and Eps8 protein levels in A549 and A549 + ITSN-1s cell lysates. Mouse IgG was used as control (not shown). **e** Densitometry of Eps8-mSos1 interaction expressed as percentage of control. All data are representative of 3 independent experiments performed under identical experimental conditions

Changes in cell cytoskeleton organization are mediated by the GTPase proteins and it has been shown that ITSN-1s interacts with mSos1, a GEF for Rac1, and with CdGAP, a GAP for Cdc42 [6, 22, 23, 35]. We therefore addressed whether ITSN-1s may regulate the activation status of these proteins. A549 and A549 + ITSN-1s cell lysates were subjected to Rac1, Cdc42 and RhoA activation assays (Fig. 7a). Densitometry demonstrated a 32 % decrease in activated Rac1 and 18 % increase in RhoA but no significant difference in activated Cdc42 in A549 + ITSN-1s cells compared to A549 cells (Fig. 7b). These findings are consistent with previous reports indicating a reciprocal balance between Rac1 and RhoA [42, 43].

Rac1 is activated by interacting with mSos1-Eps8 complex which is formed in the presence of activation of RTK [44]. Since ITSN-1s is an mSos1 interacting protein, we performed IP of A549 and A549 + ITSN-1s cell lysates with mSos1 Ab and WB with Rac1 Ab to evaluate if restoring ITSN protein level interferes with mSos1-Rac1 interaction. No detectable difference was seen in the mSos1-Rac1 interaction between A549 and A549 + ITSN-1s cells (Fig. 7c, upper panel). The lower panels in Fig. 7c indicate the protein level of Rac1 and mSos1 in A549 and A549 + ITSN-1s lysates. As Eps8 is the other component of the complex required for Rac1 activation, we next evaluated if the level of ITSN protein level impacted mSos1-Eps8 interaction. IP using mSos1 Ab showed less mSos1-Eps8 interaction in A549 + ITSN-1s cell lysates compared to A549 cells (Fig. 7d, upper panel). Moreover, WB of cell lysates revealed that the level of Eps8 protein is lower in A549 + ITSN-1s compared to A549 lysates, while the level of mSos1 protein is unchanged (Fig. 7d, lower panels). Densitometry indicated that restoring ITSN-1s decreased Eps8 protein level by 20 % and impaired the effective formation of mSos1-Eps8 complex by 54 % (Fig. 7e).

### ITSN-1s enhances Cbl-Eps8 interaction leading to increased ubiquitination of Eps8

**Fig. 8:**
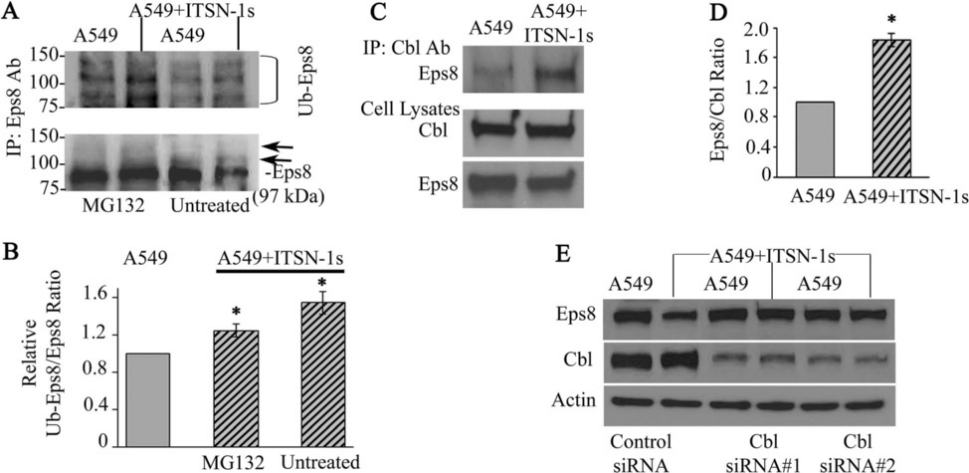
ITSN-1s enhances Cbl-Eps8 interaction leading increased ubiquitination of Eps8. **a** A549 and A549 + ITSN-1s cells pretreated with MG132 (10 μM for 2 h) and untreated cells were lysed and subjected to IP with Eps8 Ab followed by WB with Eps8 and Ub Abs. Arrows and bracket show ubiquitinated Eps8. **b** Densitometry of Ub-Eps8 expressed as percentage of control. **c** IP with Cbl Ab followed by WB with Eps8 Ab. The lower panels illustrate the Cbl and Eps8 protein levels in A549 and A549 + ITSN-1s cell lysates. Rabbit IgG was used as control (not shown). **d** Densitometry of Eps8-Cbl interaction expressed as percentage of control. **e** A549 and A549 + ITSN-1s cells transfected with Cbl-siRNA#1 and #2 or control-siRNA. Lysates were blotted with Cbl Ab (middle panel) followed by Eps8 Ab (*upper panel*). Actin was used as loading control (*lower panel*). Data are shown as mean ± SE. *p < 0.05; All data are representative of 3 independent experiments performed under identical experimental conditions

ITSN-1s is known to interact with Cbl and enhance its activity leading to ubiquitination of important signaling proteins [7, 8]. Therefore, we investigated if decreased Eps8 protein level in A549 + ITSN-1s is due to its ubiquitination and degradation. Cells pretreated with 10 μM of MG132 (Sigma-Aldrich, St. Louis, MO) for 2 h and untreated cells were lysed and IP performed with Eps8 Ab, followed by WB with both Eps8 (Fig. 8a, lower panel) and ubiquitin (Fig. 8a, upper panel) Abs. MG132 is a specific and potent proteasome inhibitor which reduces the degradation of ubiquitin-conjugated proteins [45]. Untreated A549 + ITSN-1s cells demonstrated less full-length Eps8 compared to A549 cells and additional bands above (Fig. 8a, arrows), which represent the ubiquitinated Eps8 protein. The same protein bands were immunoreactive to ubiquitin Ab (Fig. 8a, bracket). Pretreatment with MG132 resulted in restoration of Eps8 protein level (Fig. 8a, lower panel) and marked accumulation of ubiquitinated Eps8 (Fig. 8a, upper panel), indicating that ITSN-1s mediated downregulation of Eps8 is via proteasomal degradation. In the presence of MG132, A549 + ITSN-1s cells showed relatively limited accumulation of ubiquitinated Eps8 when compared to A549 cells (Fig. 8b). To confirm that Eps8 ubiquitination is via Cbl, we performed IP with Cbl Ab and WB with Eps8 Ab (Fig. 8c) which demonstrated increased interaction between Cbl and Eps8 in A549+ ITSN-1s cells (Fig. 8c, upper panel). The lower panels in Fig. 8c indicate the level of Cbl and Eps8 in the A549 and A549 + ITSN-1s lysates. Densitometry analyses indicate that ITSN restoration improved the effective formation of Cbl-Eps8 complex by 83 % (Fig. 8d). To further validate that Eps8 degradation is via Cbl, we used a siRNA approach to knockdown Cbl protein level. A549 and A549 + ITSN-1s cells were transfected with Cbl-siRNA and control-siRNA (Fig. 8e). Cbl protein level was unaffected by control-siRNA, whereas Cbl-siRNA efficiently knocked down the Cbl protein level in A549 and A549 + ITSN-1s cells (Fig. 8e, middle panel). Moreover, transfection with control-siRNA showed degradation of Eps8 in A549 + ITSN-1s cells (Fig. 8e, upper panel-left 2 lanes). However, with Cbl knockdown, Eps8 degradation in A549 + ITSN-1s cells was prevented (Fig. 8e, upper panel-right 4 lanes).

**Fig. 9:**
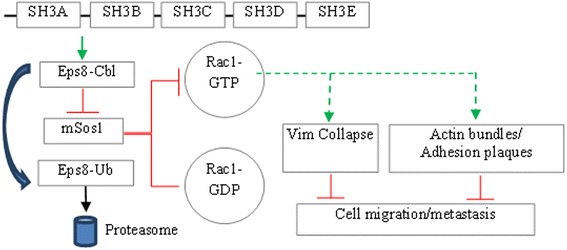
Proposed mechanism for ITSN-1s-mediated Eps8/Cbl/Sos1 interaction and Rac1-dependent LC cell migration and metastasis. ITSN-1s enhances Cbl-Eps8 interaction leading to ubiquitination and degradation of Eps8. The preferential formation of Cbl-Eps8 leads to impaired assembly of Sos1-Eps8, leading to deactivation of Rac1. Rac1 deactivation reorganizes the cytoskeleton structure to increase thick actin bundles, increase focal adhesion complexes and collapse of the vimentin filament network altogether leading to decreased LC cell migration and metastasis

In summary, our studies show that restoring ITSN-1s increases Cbl-Eps8 interaction leading to Eps8 ubiquitination and degradation. The preferential formation of Cbl-Eps8 contributes to impaired assembly of Eps8-mSos1 complex, leading to Rac1 inactivation, reorganization of actin into thick bundles, increased FA complexes and collapse of the vimentin filament network altogether leading to decreased LC cell migration and metastasis (Fig. 9).

## Discussion

Our study is the first to investigate the role of ITSN-1s in LC. We found that ITSN-1s level is consistently low in all LC tissues and LC cell lines. Based on our pilot data demonstrating a negative correlation between ITSN-1s level and the aggressiveness of LC, further studies will be performed to evaluate the use of ITSN-1s as a biomarker to predict the aggressiveness and prognosis of LC. The goal of this study was to evaluate the mechanisms involved, and the impact of ITSN-1s deficiency in LC progression. Our studies demonstrate that ITSN-1s deficiency plays a significant role in all the key stages of LC – cell proliferation, anchorage-independent growth, migration and metastasis. These findings are consistent with our previous studies using animal models of human disease and human tissue which demonstrated that chronic ITSN-1s deficiency of lung tissue triggers phenotypic changes toward proliferation [9, 16, 17]. Our findings are also consistent with the clinical evidence demonstrating a lower incidence of LC in patients with Down syndrome who overexpress ITSN-1s protein [25]. The Human Protein Atlas reports significantly lower levels of ITSN-1s level in LC and a number of other solid organ malignancies which further supports our data [46]. However, contrary to our findings, studies in neuroblastoma and glioblastoma have shown upregulation of ITSN-1s and reduced tumorigenesis and cell migration with silencing of ITSN-1s [47–49]. This demonstrates that ITSN-1s medicated interactions and regulation of signaling pathways are specific to the tissue and disease. There are a number of minor splice variants of ITSN-1s protein which facilitate tissue specific interactions. This is illustrated in the fact that ITSN-1s preferentially interacts with mSos1 and Cbl in most tissues including lung, however, in brain tissue a splicing of microexon 20 within the SH3A domain of ITSN-1s (resulting in inclusion of 5 additional amino acids) leads to reduced binding to mSos1 and Cbl and enhanced interaction with CdGAP [50].

ITSN-1s’ interaction with CdGAP and mSos1 has been shown to regulate the activity of GTPase proteins. However, ITSN-1s does not have a GEF domain like ITSN-1l (C-terminal DH-PHdomains act as a GEF). ITSN-1s’ interaction with CdGAP inhibits its GAP activity leading to activation of Cdc42 and Rac1 [22]. ITSN-1s’ interaction with mSos1 is more complex. The SH3 domains of ITSN-1s form a stable complex with mSos1 and compete with Grb2 for binding to the same site on mSos1. Consistent with this, overexpression of the SH3 domains of ITSN impairs mSos1-Grb2 complex and prevents Ras-mediated MAPK activation [6, 35]. However, overexpression of full length ITSN-1s was shown to activate Ras in the perinuclear vesicles without downstream activation of the MAPK pathway [51]. ITSN-1s mediated Ras activation in the perinuclear vesicles was initially thought to be via mSos1 but recent studies have disproved this and have implicated a novel PI3K isoform in this interaction [49]. Given the wide subcellular distribution of ITSN-1s, it is not surprising that ITSN-1s has unique interactions and regulates different signaling pathways depending on the intracellular compartment and its spatial orientation.

The role of ITSN-1s in the regulation of cell migration and the underlying mechanism has not been previously reported. It is well-known that Eps8 interacts with mSos1 in the presence of RTK activation to convert the Rac1 GTPase from its inactive GDP-bound state to the active GTP-bound state [20]. The role of ITSN-1s in mSos1 GEF activity towards Rac1 has not been previously reported. Our studies demonstrate that full length ITSN-1s impairs mSos1-Eps8 interaction and favors Cbl-Eps8 interaction leading to impaired Rac1 activation and Eps8 ubiquitination respectively. E3 ubiquitin ligases, especially Cbl, play an important role in regulating the level of numerous proteins [52]. Mutations and deregulation of Cbl are highly prevalent in LC [19]. Herein, for the first time, we have shown that Eps8 protein level is regulated by Cbl and have demonstrated that ITSN-1s is an important mediator of this interaction. We predicted the interaction is most likely between the SH3 domain(s) of ITSN and the proline-rich regions of Eps8 and Cbl, similar to ITSN’s interactions with most signaling proteins [53]. Our additional studies (unpublished) indeed demonstrate direct interaction between ITSN-1s’ SH3 domains and Eps8. Our work in progress aims to identify the specific SH3 domain(s) of ITSN-1s involved, and map the specific region of the domain involved in the interaction. Prior study by Ding et al. reported direct interaction between ITSN-2 and Eps8 but via the CC region [54]. Although the CC region is one of the less conserved regions between ITSN-1 and ITSN-2 [15], in future studies we will explore if there is a role for the ITSN-1s’ CC region in the regulation of Eps8. The multiple domains of ITSN-1s are known to simultaneously interact with the same protein as well as different proteins [55]. Given the presence of 5 SH3 domains, it is likely that ITSN-1s is able to simultaneously interact with multiple proline-rich domains of proteins to coordinate different cellular signaling processes. ITSN deficiency leading to impaired Cbl-Eps8 interaction, and enhanced mSos1-Eps8 complex formation leading to Rac1 activation, is a mechanism which has not been previously reported in cancer. ITSN’s impact on EGFR protein level and potential impact on mSos1-Eps8 interaction and Eps8 ubiquitination cannot be ruled out.

Recognizing ITSN-1s deficiency as a key player in Rac1 activation is significant since Rac1 is the most important regulator of the cytoskeleton structure with control over epithelial-mesenchymal transition, cell migration and metastasis of LC [56]. The other GTPases, Cdc42 and Rho, are not able to drive cell migration efficiently in the absence of Rac1 [57]. Rac1 has a complex reciprocal relationship with RhoA, and the balance between the two determines cell migration by reorganizing cytoskeleton elements and focal adhesions [43, 58]. Consistent with this, our studies showed decreased Rac1 activation, increased RhoA activation and significant cytoskeleton modification with ITSN-1s restoration. Compared to A549 cells, A549 + ITSN-1s cells showed increased spreading, lack of elongated and polarized morphology and prominent actin bundles towards peripheral attachment points. These findings are consistent with previous observations in Rac1 deficient cells [57] and are likely due to a change from a mesenchymal to amoeboid type of cell migration seen with decreased Rac1 and activation of RhoA [58]. In addition, we noted an increase in the number of vinculin focal adhesions at the cell surface of A549 + ITSN-1s cells. Vinculin deficient cells have impairment of traction, spreading and ECM adhesion all leading to increased cell motility [39]. Activated vinculin binds to integrin and cadherin and mediates cell-ECM and cell-cell junctions to stabilize cells. Upregulation of vinculin increases E-cadherin (epithelial marker) and downregulation increases vimentin (mesenchymal marker) [59]. Consistent with this concept, the wide subcellular distribution of vimentin filaments present in A549 cells was collapsed in the perinuclear area in A549 + ITSN-1s cells. The impaired formation of vimentin filaments in A549 + ITSN-1s cells could also be due to impaired PAK activation as a result of decreased Rac1 activation [60]. Besides Rac1, there may be other proteins and signaling pathways involved in cytoskeleton regulation, possibly impacted by regulation of ITSN-1s level, but not explored in this study. As an actin-capping protein, it is possible that in addition to its effects via Rac1, Eps8 may also have directly contributed to changes to the actin cytoskeleton [21].

Our findings of decrease in cell proliferation, in anchorage-independent growth and in tumor growth with restoration of ITSN-1s protein level is contrary to previous studies showing increased Ras activation which is known to drive cell proliferation and tumorigenesis [61]. However, studies have shown that Rac1 activation is a pre-requisite for Ras-mediated tumor progression [62]. Rac1 activation could also directly activate JNK pathway leading to tumor progression [63]. As an oncoprotein, Eps8 also translocates to the nucleus and upregulates numerous cell cycle proteins such as transcription factor FOXM1 [64]. Therefore, ITSN-1s mediated inhibition of tumorigenesis is likely due to a combined inhibitory effect on these pathways. In addition, ITSN-1s enhances ubiquitination and downregulation of EGFR with a potential negative impact on proliferation [7].

## Conclusion

We demonstrate that restoring ITSN-1s protein level impairs both proliferation and anchorage-independent growth, and restores cytoskeleton changes in favor of decreased cell migration and metastasis. Based on these results, we propose a novel mechanism of LC regulation triggered by ITSN-1s deficiency, consistent with the idea that low ITSN-1s decreases Eps8-Cbl interaction and enhances the assembly of Eps8-mSos1 complex, leading to upregulation of Eps8 and activation of Rac1. ITSN-1s’ ability to reverse the malignant features demonstrates the capability of this protein to regulate multiple pathways simultaneously which makes it an attractive therapeutic target. Further validation of ITSN-1s protein level in a large cohort of patients at different stages of LC could establish ITSN-1s as a predictor of prognosis and indicator of response to therapy. Given the ubiquitous distribution of ITSN-1s and evidence that loss of ITSN-1s is a characteristic feature of many cancers [46], our findings may be applicable to other types of cancer.

## Additional files

Additional file 1: Figure S1. ITSN-1s impairs LC cell migration. (A) A549 and A549 + ITSN-1s cells were grown to confluence in 3 sets in triplicate - 1 set of cells were trypsinized and counted (controls), 1 set was subjected to treatment with mitomycin C (7.5 μg/ml for 1 h) and the other set was untreated. We studied in preliminary experiments, different concentrations and duration of treatment (ranging from 5 μg/ml to 20 μg/ml and 45 min to 4 h) and determined that 7.5 μg/ml of mitomycin C treatment for 1 h provided the ideal results (>90 % inhibition of proliferation without killing the cells). The latter 2 sets were cultured for an additional 24 h. After 24 h all cells were trypsinized and counted. The following formula was used to calculate cell proliferation for mitomycin C treated and untreated cells: (Average cell count of set – Average cell count of control)/Average cell count of control × 100 %. The results are reported as percentage of A549 untreated cells. Error bar represents the mean ± SE; ***p* < 0.01. Data are representative of 3 independent experiments performed under identical experimental conditions. (B) A549 and A549 + ITSN-1s cells were grown to confluence in 2 sets in triplicate, 1 set was subjected to treatment with mitomycin C (7.5 μg/ml for 1 h) and the other set was untreated. Mitomycin C treated and untreated A549 and A549 + ITSN-1s cell layer was wounded using a sterile micropipette tip; the area was examined after 24 h with phase contrast microscopy. Data are representative of 3 independent experiments performed under identical experimental conditions. (TIF 2992 kb) Additional file 2: Figure S2. ITSN-1s expression in emerging tumors. Immunofluorescent staining with ITSN-1 Ab followed by AF488 Ab of lung sections from mice injected retro-orbitally with A549 (A, C) and A549 + ITSN-1s (B, D) cells. Representative images of small tumors (A, B, b1) and medium/large tumors (C, D, d1) in mice injected with A549 and A549 + ITSN-1s cells respectively. 3 sections per mice (*n* = 3 mice) were examined and images were acquired using identical parameters. Bars: 40 μm. (TIF 3224 kb)

## Abbreviations

A549 + ITSN-1s: myc-ITSN-1s transfected A549 cells
Ab: Antibody
AF: Alexa Fluor
CdGAP: GTPase activating protein
ECM: Extracellular matrix
FA: Focal adhesion
GEF: Guanine exchange factor
H&E: Hematoxylin and eosin
IHC: Immunohistochemistry
IP: Immunoprecipitation
ITSN-1s: Intersectin-1s
LC: Lung cancer
PAK: p21 activated kinase
RT: Room temperature
RTK: Receptor tyrosine kinases
TMA: Tissue micro array
Ub: Ubiquitin
WB: Western blot
