# Supplementary figures and images for: Rac1-mediated cytoskeleton rearrangements induced by intersectin-1s deficiency promotes lung cancer cell proliferation, migration and metastasis
Source: Mol Cancer. 2016 Sep 14;15:59. doi: 10.1186/s12943-016-0543-1 (PMC5024437; doi:10.1186/s12943-016-0543-1)

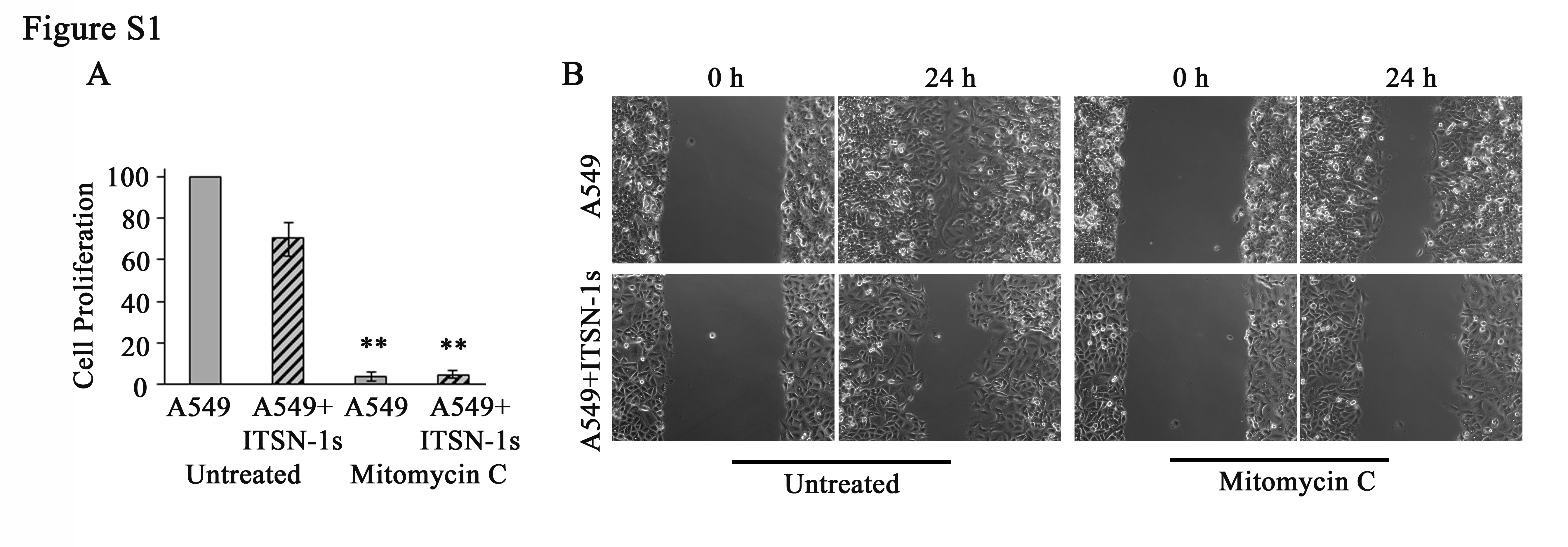

Supplement: Additional file 1: Figure S1. — ITSN-1s impairs LC cell migration. (A) A549 and A549 + ITSN-1s cells were grown to confluence in 3 sets in triplicate - 1 set of cells were trypsinized and counted (controls), 1 set was subjected to treatment with mitomycin C (7.5 μg/ml for 1 h) and the other set was untreated. We studied in preliminary experiments, different concentrations and duration of treatment (ranging from 5 μg/ml to 20 μg/ml and 45 min to 4 h) and determined that 7.5 μg/ml of mitomycin C treatment for 1 h provided the ideal results (>90 % inhibition of proliferation without killing the cells). The latter 2 sets were cultured for an additional 24 h. After 24 h all cells were trypsinized and counted. The following formula was used to calculate cell proliferation for mitomycin C treated and untreated cells: (Average cell count of set – Average cell count of control)/Average cell count of control × 100 %. The results are reported as percentage of A549 untreated cells. Error bar represents the mean ± SE; **p < 0.01. Data are representative of 3 independent experiments performed under identical experimental conditions. (B) A549 and A549 + ITSN-1s cells were grown to confluence in 2 sets in triplicate, 1 set was subjected to treatment with mitomycin C (7.5 μg/ml for 1 h) and the other set was untreated. Mitomycin C treated and untreated A549 and A549 + ITSN-1s cell layer was wounded using a sterile micropipette tip; the area was examined after 24 h with phase contrast microscopy. Data are representative of 3 independent experiments performed under identical experimental conditions. (TIF 2992 kb) [file 12943_2016_543_MOESM1_ESM.tif]

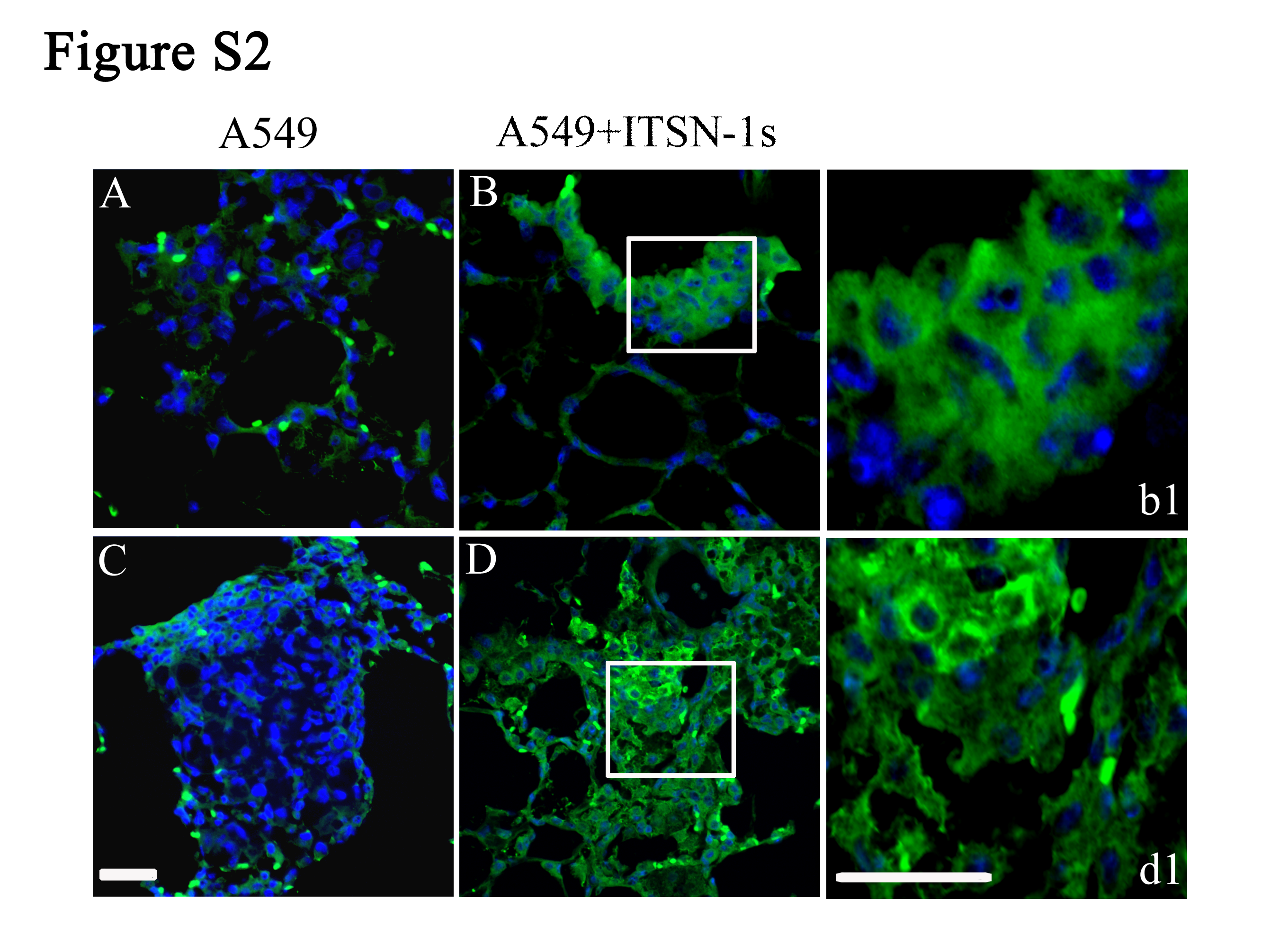

Supplement: Additional file 2: Figure S2. — ITSN-1s expression in emerging tumors. Immunofluorescent staining with ITSN-1 Ab followed by AF488 Ab of lung sections from mice injected retro-orbitally with A549 (A, C) and A549 + ITSN-1s (B, D) cells. Representative images of small tumors (A, B, b1) and medium/large tumors (C, D, d1) in mice injected with A549 and A549 + ITSN-1s cells respectively. 3 sections per mice (n = 3 mice) were examined and images were acquired using identical parameters. Bars: 40 μm. (TIF 3224 kb) [file 12943_2016_543_MOESM2_ESM.tif]
